# Cobalt Oxide Nanosheet and CNT Micro Carbon Monoxide Sensor Integrated with Readout Circuit on Chip

**DOI:** 10.3390/s100301753

**Published:** 2010-03-03

**Authors:** Ching-Liang Dai, Yen-Chi Chen, Chyan-Chyi Wu, Chin-Fu Kuo

**Affiliations:** 1 Department of Mechanical Engineering, National Chung Hsing University, Taichung, 402 Taiwan; E-Mail: metallica0515@msn.com (Y.-C.C.); 2 Department of Mechanical and Electro-Mechanical Engineering, Tamkang University, Tamsui, 251 Taiwan; E-Mail: ccwu@mail.tku.edu.tw (C.-C.W.); 3 Microsystems Technology Center, Industrial Technology Research Institute, Tainan, 709 Taiwan; E-Mail: kuochinfu@itri.org.tw (C.-F.K.)

**Keywords:** micro carbon monoxide sensor, cobalt oxide film, readout circuit

## Abstract

The study presents a micro carbon monoxide (CO) sensor integrated with a readout circuit-on-a-chip manufactured by the commercial 0.35 μm complementary metal oxide semiconductor (CMOS) process and a post-process. The sensing film of the sensor is a composite cobalt oxide nanosheet and carbon nanotube (CoOOH/CNT) film that is prepared by a precipitation-oxidation method. The structure of the CO sensor is composed of a polysilicon resistor and a sensing film. The sensor, which is of a resistive type, changes its resistance when the sensing film adsorbs or desorbs CO gas. The readout circuit is used to convert the sensor resistance into the voltage output. The post-processing of the sensor includes etching the sacrificial layers and coating the sensing film. The advantages of the sensor include room temperature operation, short response/recovery times and easy post-processing. Experimental results show that the sensitivity of the CO sensor is about 0.19 mV/ppm, and the response and recovery times are 23 s and 34 s for 200 ppm CO, respectively.

## 1. Introduction

Carbon monoxide sensors are widely applied in industrial and environmental monitoring. The sensitivity of gas sensors depends on the material of their sensing films. Several materials, such as SnO_2_, ZnO, ZnO-CuO, In_2_O_3_ and WO_3_-In_2_O_3_ [1–5], have been utilized as CO gas sensing films. However, the above-mentioned films share as a common weakness that the working temperature is too high, leading to increased power consumption. Cobalt oxide can sense CO gas at room temperature [6]. Wu *et al.* [7] manufactured a CO sensor based on the sensing material CoOOH-WO_3_ with single wall CNTs. The role of the added CNTs was to act as a conducting wire and increase the electric conductivity of the sensing film, resulting in shortened response and recovery times for the sensor. Consequently, in this study cobalt oxide with CNTs was adopted as a CO sensing material.

Several micro devices have been manufactured using microelectromechanical system (MEMS) technology [8]. Micro gas sensors fabricated by MEMS technology have the advantages of small size, high performance, low cost and easy mass-production. Many studies have used MEMS technology to develop micro carbon monoxide sensors. For instance, Tabata *et al.* [9] proposed a micro CO sensor manufactured using a silicon micromachining technique; its structure consisted of a catalytic thick film/SnO_2_ thin film bilayer and a thin film heater. The thin film heater and the SnO_2_ thin film sensing layer were deposited on a silicon oxide-silicon nitride membrane. Chan *et al.* [10] reported a gas sensor with a micro hotplate for CO sensing, and the thermally isolated hotplate was fabricated using a surface silicon micromachining technique. Barrettino *et al.* [11] fabricated a microsystem for gas detection using the industrial 0.8 μm CMOS technology combined with post-CMOS micromachining. The microsystem comprised an array of three micro hotplates, and three single-ended temperature controllers were used to regulate the micro hotplate temperature up to 350°C. The micro hotplates were covered with tin dioxide for CO gas sensing. Briand *et al.* [12] presented a gas sensor with micromachined hotplates for CO gas sensing applications. The sensor was coated with a Pd-doped tin oxide drop and annealed using the integrated heater. These CO sensors [9–12] did not have integrated circuits-on-a-chip, so they needed to couple with circuits by packaging, leading to an increase in package cost. Integrating gas sensors with circuits-on-a-chip helps to reduce the packaging cost and enhance the performance. Thereby, in this work a carbon monoxide sensor integrated with a readout circuit-on-a-chip was developed. The readout circuit is an instrumentation amplifier circuitry [13] that can convert the sensor resistance into an output voltage.

The manufacturing technique which uses the commercial CMOS process to fabricate MEMS devices is called CMOS-MEMS [14–16]. Micro devices made by the CMOS-MEMS technique usually need a post-process to coat the functional films [17] or to release the suspended structures [18]. For example, Liu *et al.* [17] coated a sensitive film of polyaniline nanofiber on a micro ammonia sensor using a post-process. The benefits of CMOS-MEMS micro devices include integration with integrated circuits-on-a-chip, low cost per unit area and easy mass-production utilizing semiconductor foundries. In this study, the CMOS-MEMS technique was employed to fabricate a micro carbon monoxide sensor integrated with a readout circuit-on-a-chip. The sensing film of the sensor is a composite cobalt oxide nanosheet and carbon nanotube film, which was synthesized by a precipitation-oxidation method. The method needs to add a precipitant and an oxidant into the synthesized material. For instance, Wu *et al.* [19] used a precipitation-oxidation method to prepare a cobalt oxide film that was made from a Co(NO_3_)_2_ solution via precipitation with NaOH and oxidation in air. The carbon monoxide sensor needs post-processing to coat the sensing film. The post-process employed etchants to etch the sacrificial layers, and then the sensing film is coated on the sensor. When the sensing film adsorbs or desorbs CO gas, the sensor generates a change in resistance. The resistance of the sensor was converted by the readout circuit into the voltage output.

## 2. Preparation of the CO Sensing Film

The CO sensing film, a composite film of cobalt oxide and carbon nanotubes, was synthesized by the precipitation-oxidation method [20]. In the procedure, 1.5 g cobaltous nitrate [Co(NO_3_)_2_·6H_2_O] was dissolved in 50 mL DI water with vigorous stirring. Next, aqueous sodium hydroxide (NaOH) solution (5M, 100 mL) was added drop-wise to the above solution under constant stirring until the pH of the suspension increased to 12, then 1 mL of CNT solution was added to the mixed solution, as shown in Table 1. Then, the flask was sealed and heated at 80 °C for 24 h. After the reaction was completed, the solution needed to cool to room temperature naturally. The precipitate was washed with DI water and ethanol. Finally, the film was coated on the silicon substrate, followed by calcination in air at 120 °C for 2 h.

**Figure 1.**
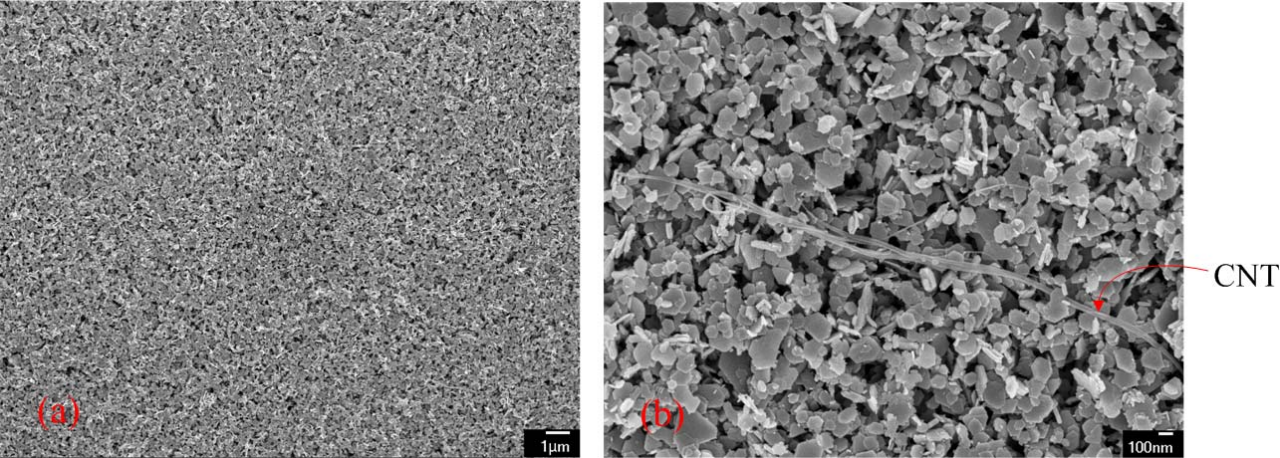
SEM images of the CoOOH/CNT film; (a) low magnification; (b) high magnification.

**Figure 2.**
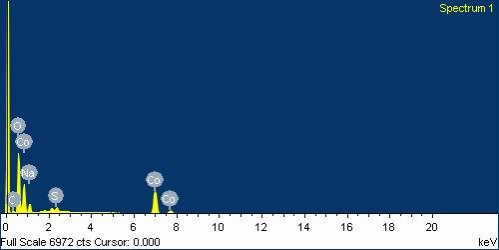
Elements of CoOOH/CNT film measured by energy dispersive spectrometer

The surface morphology of the CoOOH/CNT film was examined by scanning electron microscopy (SEM) (JEOL JSM-6700F). Figures 1(a) and (b) show the low- and high-magnification scanning electron microscopy images of the CoOOH/CNT film after the synthesis. Figure 1(a) shows that the film has a porous structure. Figure 1(b) indicates that the film consists of a CoOOH nanosheet and CNTs. The elements of the CoOOH/CNT film were measured by an energy dispersive spectrometer (OXFORD INCA ENERGY 400), and the measured results are shown in Figure 2. The CoOOH/CNT film contained 43.5 wt% Co, 42.8 wt% O, 6.9 wt% C, 5.5 wt% Na and 1.3 wt% S. The composition of the film is summarized in Table 2. The main components of the film were cobalt and oxygen. The element Na resulted from the precipitant of NaOH, and the element S was generated by the dispersant of sodium dodecyl sulfate in the CNT solution. The influence of these elements on the sensing film was assumed to be negligible dues to the very small amounts present.

## 3. Structure of the CO Sensor

**Figure 3.**
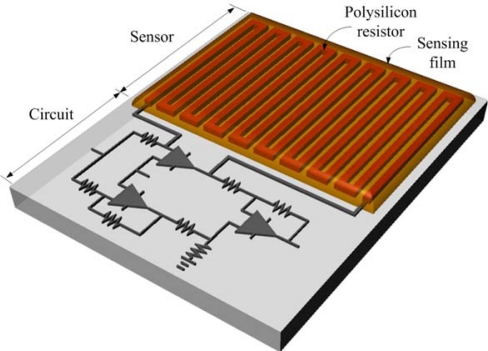
Schematic structure of the CO sensor with the readout circuit.

Figure 3 illustrates the structure of the integrated chip that contains a CO sensor and a readout circuit. The area of the CO sensor is about 1 mm^2^. The CO sensor consists of a polysilicon resistor and a CO sensing film. A silicon dioxide layer is located between the polysilicon resistor and the sensing film. As shown in Figure 3, the polysilicon resistor is connected to the readout circuit. The CoOOH/CNTs CO sensing film is coated on the polysilicon resistor. The polysilicon resistor is 2 μm wide, 0.4 μm thick and 11,000 μm long. When the sensing film of the sensor absorbs or desorbs CO gas, its energy band produces a change, resulting in changes to the energy band of the polysilicon resistor [21]. The polysilicon resistor generates a change in resistance as its energy band varies. The resistance variation of the CO sensor is converted by the readout circuit into the voltage output.

**Figure 4.**
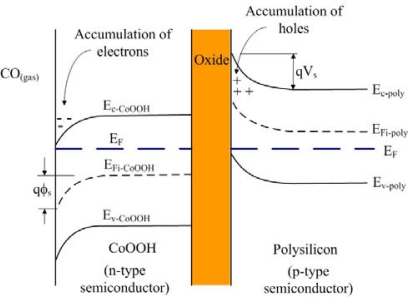
Energy band diagram of the CO sensor. E_F_ is the Fermi level, E_c-CoOOH_ is the conduction band of CoOOH, E_v- CoOOH_ is the valence band of CoOOH, E_Fi-CoOOH_ is the intrinsic Fermi level of CoOOH, E_c-poly_ is the conduction band of polysilicon, E_v-poly_ is the valence band of polysilicon, E_Fi-poly_ is the intrinsic Fermi of polysilicon, *q* is the electronic charge, *ϕ_s_* is negative surface potential and *V_s_* is the potential barrier.

The CO sensing mechanism on CoOOH has been reported to take the form of gas-phase CO adsorption and desorption on cobalt sites, and reaction of the adsorbed CO with lattice oxygen atoms to form CO_2_ [19]. Equation (1) represents the adsorption and desorption of CO and Equation (2) shows the surface reaction of CO and O_2_:

(1) $$\text{CO}+*+e^{-}↔\text{CO}-{*}^{-}$$

(2) $$\text{CO}-{*}^{-}+O-*\to {\text{CO}}_{2}+2*+e^{-}$$

where * represents the active sensing vacant sites on the surface and CO–*^−^ is the absorbed CO on the surface. Figure 4 illustrates the energy band diagram of the CO sensor. The cobalt oxide is an n-type semiconductor, and the polysilicon is p-type. When the surface of CoOOH is exposed to CO gas, electrons are produced at the surface of CoOOH according to Equation (2). As shown in Figure 4, an accumulation of electrons is formed at the surface of CoOOH when CO gas interacts with CoOOH, so that the conduction and valence band edges of CoOOH bend downward, resulting in generation of a negative surface potential, *ϕ_s_*. An accumulation of holes at the oxide-polysilicon interface is formed by the negative surface potential *ϕ_s_*, which leads to the conduction and valence band edges of polysilicon to bend upward and causes the production of the potential barrier, *V_s_* [21]. When the CoOOH film of the CO sensor is in a high CO environment, the surface potential *ϕ_s_* increases, resulting in an increase of the potential barrier *V_s_* and a decrease of the polysilicon resistance. Therefore, the resistance of polysilicon increases as the amount of CO sensed by the CoOOH increases.

**Figure 5.**
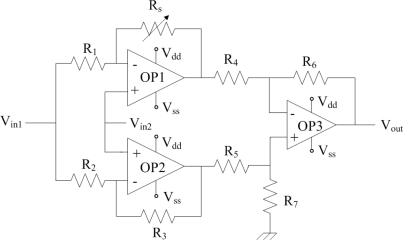
Readout circuit for the CO sensor.

Figure 5 illustrates the readout circuit for the CO sensor, where *R_s_* represents the resistance of the sensor; *V_dd_* is the bias voltage of the circuit; *V_ss_* is the ground; *V_in1_* and *V_in2_* are the input voltages of the circuit and *V_out_* is the output voltage of the circuit. The readout circuit contains three amplifiers, where *OP1* and *OP2* are non-inverting amplifiers; *OP3* is a difference amplifier. The resistance of the sensor changes as the sensing film adsorbs or desorbs CO gas. The readout circuit is employed to convert the resistance of the CO sensor into the voltage output. In this design, *R_1_*=1 kΩ, *R_2_*=1 kΩ, *R_3_*=10 kΩ, *R_4_*=100 Ω, *R_5_*=100 Ω, *R_6_*=15 kΩ and *R_7_*=20 kΩ are adopted. HSPICE, which is a professional circuit simulation software, is utilized to simulate the characteristics of the readout circuit.

**Figure 6.**
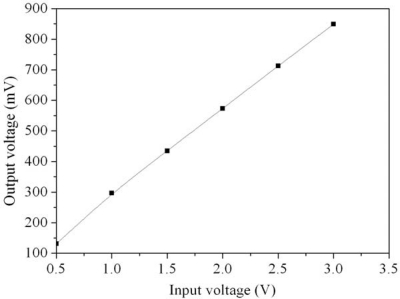
Relation between the output and input voltages for the readout circuit.

Figure 6 shows the relation between the input voltage *V_in1_* and the output voltage *V_out_* for the readout circuit. In this simulation, the resistance *R_s_* of the sensor is set as 20 kΩ. The bias voltage *V_dd_* is 3.3 V and the input voltage *V_in2_* is 3 V. The simulated results depict that the output voltages of the readout circuit are 300, 570 and 850 mV when the input voltages *V_in1_* are 1, 2 and 3 V, respectively. The input voltage of the readout circuit increases, then its output voltage becomes large.

**Figure 7.**
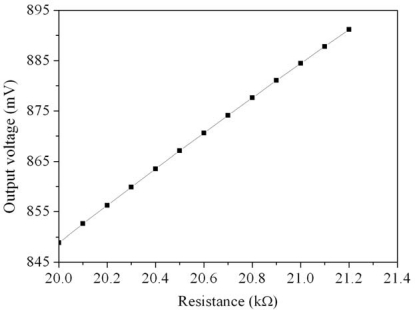
Simulated results of the output voltage for the CO sensor.

Figure 7 presents the relation between the output voltage of readout circuit and the resistance of sensor. In this investigation, the input voltages *V_in1_* and *V_in2_* are 3 V, the resistance of the sensor changes from 20 to 21.2 kΩ. The output voltage of the readout circuit changes from 850 to 890 mV as the resistance of the sensor varies from 20 to 21.2 kΩ.

## 4. Fabrication of the CO Sensor

**Figure 8.**
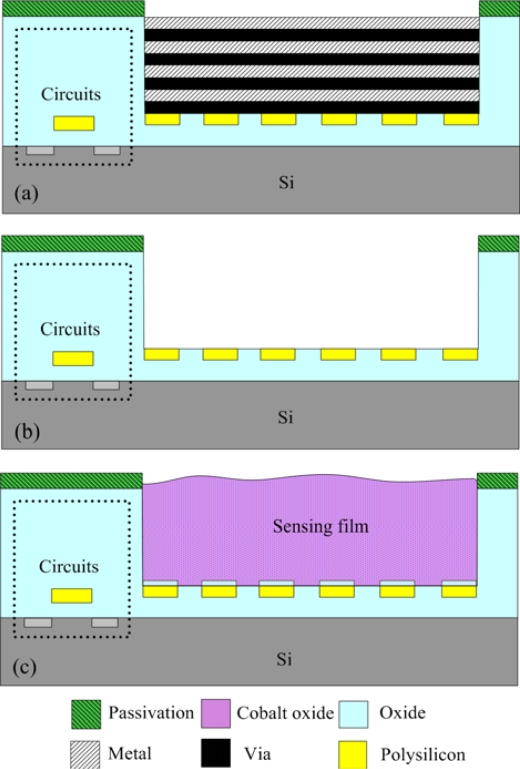
Fabrication process of the CO sensor: (a) after the CMOS process, (b) etching the sacrificial layer, (c) coating the sensing film.

**Figure 9.**
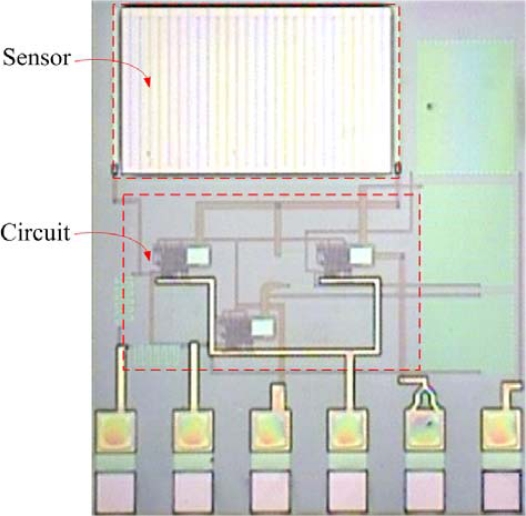
Photographic image of the CO sensor after the CMOS process.

The micro CO sensor integrated with a readout circuit-on-a-chip was fabricated using the commercial 0.35 μm CMOS process of the Taiwan Semiconductor Manufacturing Company (TSMC). After completion of the CMOS process, the CO sensor chip needs a post-process to expose the polysilicon resistor and coat the CO sensing film. The post-process consisted of two main steps: (1) the sacrificial layers were etched to expose the polysilicon resistor; (2) the CO sensing film was coated on the polysilicon resistor. Figure 8 illustrates the fabrication process of the CO sensor chip. Figure 8(a) presents the cross-section of the CO sensor chip after completion of the CMOS process. Figure 9 depicts a photograph of the CO sensor with its readout circuit after the CMOS process.

**Figure 10.**
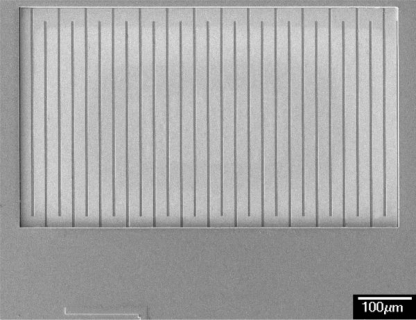
SEM image of the CO sensor after the wet etching process.

In the CO sensor, the sacrificial layers were the metal and via layers. The materials of the via and metal layers were tungsten (W) and aluminum (Al), respectively. The sacrificial layers have to be removed from the sensor chip, exposing the polysilicon resistor. As shown in Figure 8(b), the sensor chip was immersed in two etchants: one was an Al etchant with phosphoric acid, nitric acid, acetic acid and deionized water in a ratio of 14:1:2:3. The other was a W etchant with sulfuric acid and hydrogen peroxide in a ratio of 2:1. Figure 10 depicts a SEM image of the CO sensor after the wet etching process. Then, the sensor chip was put in an oven at 300°C for 8 h, so that a thin silicon dioxide layer was formed on the surface of polysilicon resistor. Finally, as shown in Figure 8(c), the cobalt oxide film was coated on the polysilicon resistor.

## 5. Results and Discussion

**Figure 11.**
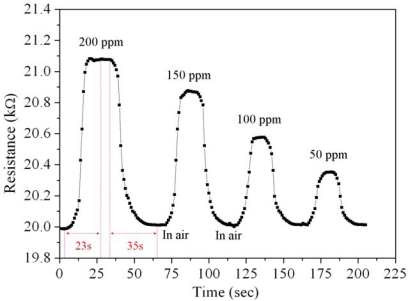
Relation between the resistance variation and CO concentration for the CO sensor.

The characteristic of the CO sensor chip was measured using a test chamber, a power supply, an LCR meter and an oscilloscope. In order to characterize the variation of resistance in the sensing part, the CO sensor was tested without the readout circuit. The CO sensor chip without readout circuit was set in the test chamber, and the LCR meter was employed to measure its resistance variation at room temperature at different CO concentrations. Figure 11 shows the results. The initial resistance of the CO sensor was about 20 kΩ (in air), and the resistance of the sensor varied to 21.1 kΩ at 200 ppm CO. The results revealed that the resistance of the CO sensor increased as the concentration of CO increased. The response time represented the reaction time of 90% from the initial resistance to the stable resistance, and the recovery time was the return time of 90% from the stable resistance to the initial resistance. As shown in Figure 11, the CO sensor had a response time of about 23 s at 200 ppm CO and a recovery time of 35 s at 200 ppm CO.

**Figure 12.**
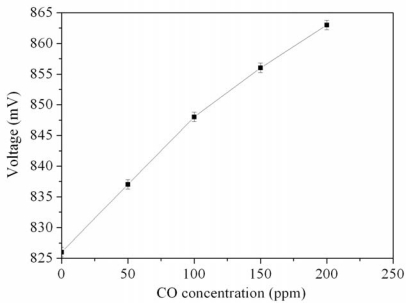
Measured results of the output voltage for the CO sensors.

The CO sensor with readout circuit was set in the test chamber and was tested at room temperature with different CO concentrations. The power supply provided a bias voltage of 3.3 V and an input voltage of 3 V to the readout circuit in the sensor. The oscilloscope was utilized to measure the output voltage of the sensor at room temperature at different CO concentrations. Figure 12 depicts the measured results of output voltage for the CO sensor with readout circuit. In this measurement, the CO gas was provided from 0 to 200 ppm. The results showed that the output voltage of the CO sensor changed from 826 to 863 mV as the concentration of CO gas varied from 0 to 200 ppm. The variation of the output voltage was 37 mV in 0–200 ppm CO. Therefore, the integrated CO sensor had a sensitivity of 0.19 mV/ppm when providing a bias voltage of 3.3 V and an input voltage of 3V.

The carbon monoxide sensors, proposed by Tabata *et al.* [9], Chan *et al.* [10], Barrettino *et al.* [11] and Briand *et al.* [12], needed micro hotplates or micro heaters to provide a high working temperature for the gas sensing films, leading to increased power consumption of the sensors. In this study, the CO sensor did not require a micro heater and could work at room temperature. These sensors [9–12] also did not have integrated circuits-on-a-chip, so they needed to use packaging to combine with circuits. In this work, the CO sensor was integrated with a readout circuit-on-a-chip using the CMOS-MEMS technique, so that the production cost was reduced. The experimental results showed that the readout circuit could operate normally after the post-process, indicat8ing that the post-process used was compatible with the commercial CMOS process.

## 6. Conclusions

A micro carbon monoxide sensor integrated with a readout circuit-on-a-chip has successfully been implemented using a commercial CMOS process and a post-process. The sensor was constructed with a polysilicon resistor and a sensing film. The sensing film of the sensor was a composite film of a cobalt oxide nanosheet and carbon nanotubes. The sensor required a post-process to coat the sensing film. The post-process utilized etchants to etch the sacrificial layers to expose the polysilicon resistor, and then the sensing film was coated on the polysilicon resistor. The advantage of the post-process was that it is compatible with the CMOS process. The sensor was a resistive type, and its resistance generated a change when the sensing film absorbed or desorbed CO gas. The resistance variation of the gas sensor was converted by the readout circuit into the output voltage. The experimental results showed that the sensitivity of the CO gas sensor was about 0.19 mV/ppm at room temperature, and the response and recovery times were 23 s and 35 s at 200 ppm CO, respectively.

## Figures and Tables

**Table 1.** Composition of the CNT solution.

**Material**	**Weight %**
Sodium dodecyl sulfate	1
CNT	9
DI water	90
Totals	100

**Table 2.** Composition of CoOOH/CNT film.

**Element**	**Weight %**
Co	43.5
O	42.8
C	6.9
Na	5.5
S	1.3
Total:	100
